# Exploring the Host Parasitism of the Migratory Plant-Parasitic Nematode *Ditylenchus destuctor* by Expressed Sequence Tags Analysis

**DOI:** 10.1371/journal.pone.0069579

**Published:** 2013-07-29

**Authors:** Huan Peng, Bing-li Gao, Ling-an Kong, Qing Yu, Wen-kun Huang, Xu-feng He, Hai-bo Long, De-liang Peng

**Affiliations:** 1 The Key Laboratory for Biology of Insect Pests and Plant Disease, Institute of Plant Protection, Chinese Academy of Agricultural Sciences, Beijing, China; 2 Huzhou Modern Agricultural Biotechnology Innovation Center, Shanghai Institutes for Biological Sciences, Chinese Academy of Sciences, Zhejiang, China; 3 Eastern Cereal and Oilseed Research Centre, Agriculture and Agri-Food Canada, Ottawa, Ontario, Canada; 4 Key Laboratory of Pests Comprehensive Governance for Tropical Crops, Environment and Plant Protection Institute, Chinese Academy of Tropical Agricultural Science, Danzhou, China; Hospital for Sick Children, Canada

## Abstract

The potato rot nematode, *Ditylenchus destructor*, is a very destructive nematode pest on many agriculturally important crops worldwide, but the molecular characterization of its parasitism of plant has been limited. The effectors involved in nematode parasitism of plant for several sedentary endo-parasitic nematodes such as *Heterodera glycines*, *Globodera rostochiensis* and *Meloidogyne incognita* have been identified and extensively studied over the past two decades. *Ditylenchus destructor*, as a migratory plant parasitic nematode, has different feeding behavior, life cycle and host response. Comparing the transcriptome and parasitome among different types of plant-parasitic nematodes is the way to understand more fully the parasitic mechanism of plant nematodes. We undertook the approach of sequencing expressed sequence tags (ESTs) derived from a mixed stage cDNA library of *D. destructor*. This is the first study of *D. destructor* ESTs. A total of 9800 ESTs were grouped into 5008 clusters including 3606 singletons and 1402 multi-member contigs, representing a catalog of *D. destructor* genes. Implementing a bioinformatics' workflow, we found 1391 clusters have no match in the available gene database; 31 clusters only have similarities to genes identified from *D. africanus*, the most closely related species to *D. destructor*; 1991 clusters were annotated using Gene Ontology (GO); 1550 clusters were assigned enzyme commission (EC) numbers; and 1211 clusters were mapped to 181 KEGG biochemical pathways. 22 ESTs had similarities to reported nematode effectors. Interestedly, most of the effectors identified in this study are involved in host cell wall degradation or modification, such as 1,4-beta-glucanse, 1,3-beta-glucanse, pectate lyase, chitinases and expansin, or host defense suppression such as calreticulin, annexin and venom allergen-like protein. This result implies that the migratory plant-parasitic nematode *D. destructor* secrets similar effectors to those of sedentary plant nematodes. Finally we further characterized the two *D. destructor* expansin proteins.

## Introduction

Expressed sequence tags (ESTs) have proven to be one of the most rapid routes to gene discovery of any organism for which a cDNA library is available [1]. In addition, large-scale EST analysis can be used to estimate gene expression levels in specific life stages or tissues and are useful tools for annotation of genome sequences [1], [2]. EST analysis has been widely applied to study the biology of nematodes. Over 1.5 million ESTs from more than 63 species, including free-living nematodes, animal-parasitic and plant-parasitic species are available in dbEST (GenBank, 1 October 2012). To date, over 125,000 EST sequences from twenty different plant-parasitic nematodes are in dbEST. This information is of great significance for studying nematode biology, especially for the identification of effectors.

Plant-parasitic nematode effectors, defined here as proteins secreted by the nematode into the host that manipulate the host to the benefit of the pathogen, are usually expressed in the subventral or dorsal pharyngeal gland cells and then secreted into the host via the stylet [3]. More than 50 effectors have been identified from plant-parasitic nematodes, including effectors that modify cell walls, or manipulate plant cell biology and host defenses [4]. Bioinformatics approaches are widely used for identifying effectors from ESTs. This approach has been used with a wide range of nematode species [5]–[17]. In the root-knot nematode, *Meloidogyne incognita*, at least 486 proteins secreted through the stylet have been discovered [18]. Coupled with the increasing availability of plant-parasitic nematode genome sequences [12], [19]–[21] and transcriptome sequences [22]–[24], it is likely that the complete effector repertoires of plant nematode species will be uncovered in the future.

*Ditylenchus destructor* is a migratory plant-parasitic nematode. Overall, some 70 crops and weeds and a similar number of fungal species have been recorded as hosts, of which sweet potato, potato and peanut are the most important. It is a serious pest of potato tubers in Europe and North America, and was also considered as an important international quarantine pest [25], [26]. In China, *D. destructor* is a major threat to sweet potato production [11], [27]. *D. africanus* is another economically important species in the *Ditylenchus* genus and its partial ESTs had been published [11]. 4847 ESTs from mixed stages of *D. africanus* were clustered into 2596 unigenes, of which 43% did not show any similarity in to any known genes. 10 putative parasitism genes were identified. *D. africanus* was previously misidentified as *D. destructor* due to their biological and taxonomical similarities [28], [29]. However, direct molecular evidences to differentiate the two spacies is lacking.

In this study, we describe the generation and analysis of 9800 ESTs from a *D. destructor* mixed-stage library. Several putative effectors and secreted proteins are identified from this dataset by using bioinformatics approaches. The differences between *D. destructor* and *D. africanus* were also investigated. In addition, two expansin genes present in the dataset were further characterized and their expression profiles were examined by *in situ* hybridization.

## Materials and Methods

### Nematode culture, cDNA library construction and sequencing

*Ditylenchus destructor* used in this study was collected in Tongshan city Jiangsu province, China, and was cultured with *Fusarium semitectum* [30]. Nematodes were collected with sterile distilled water at 3–4 weeks after inoculation. RNA was extracted with TRIzol® Reagent (Invitrogen, Carlsbad, USA), and used to construct the cDNA library with the SMART™ cDNA Library Construction Kit (Clontech, Mountain View, USA) following the manufacturer's instructions. The resulting fragments were directionally cloned in pDNR-Lib vector. The *D. destructor* mixed stage cDNA library contained over 10^6^ primary transformants. Fifty clones were randomly selected and the lengths of their cDNA insert sequences were measured by PCR with M13F and M13R primers (Table 1). 13,237 random colonies were sequenced from the 5′ ends using M13 F at the Beijing Genomics Institute (Beijing, China). Sequences were submitted to the EST division of Genbank.

**Table 1:** Primers used in this study.

Cluster ID	Gene names	primers(5′-3′)	Application
DDC05000	endo-1,4-glucanase	CAGGCTCTGAAATGCTCATGGAA	*In situ* hybridization
		TGGCAGCGTAGTAGTGCAAAGTG	
DDC00149	pectate lyase	AAGAAGGTTCCCAAGACCATTAC	*In situ* hybridization
		TTGCATTAGCCGCTGTAGACAC	
DDC04837	venom allergen protein	TCAAATTGGCAACCGACCTTTC	*In situ* hybridization
		AGACCGCTATTCGAGTCACAAG	
DDC03578	expansin	CATCGAGGCTCAGTTGAATACGC	*In situ* hybridization
		CCCAGAGGCTCCAGCACAAGAAA	
DDC03579	expansin	CCTTTCTTGTGCTGGAGCCTCTG	*In situ* hybridization
		GATGGCAATTTGTATTGGTTGGT	
DDC03835	flp-14	TTTCGCCCTCACTTTACCCATCA	*In situ* hybridization
		GAGTCAGCAGAGCACTACTTTCG	
DDC02922	annexin	CACCACAGCCAGTGCAGAATC	*In situ* hybridization
		TGCTCAAGTTGTTGGGACAGA	
DDC04384	14-3-3b	GGGCGACTACTACCGCTACTTGG	*In situ* hybridization
		AGACTGACGCTTAATTGCCTCCA	
DDC04927	calreticulin	GTTTGCGTTGGTTGCCCTGCTC	*In situ* hybridization
		CATGGACTTTGCGGTTTGATGG	
DDC03578	expansin-like-1	GGGCTTGTGGATTGGACATCAG	3′RACE amplification
		TGTGAATCACGTGGACTTGTCA	
DDC03579	expansin-like-2	AGTTGAATAAGCCAGTTCCTGA	3′RACE amplification
		CGGAGCAACTCTTACATACTTA	
DDC03578	expansin-like-1	CCATGTCTAACGCATTTTATCT	Genomic amplification
		AACGGACTTTATTTCAGTCTACTC	
DDC03579	expansin-like-2	GGATGTCTAACGCATTTTATCT	Genomic amplification
		CATTCTACAGTCTACTCGGAATT	
	M13F	GTAAAACGACGGCCAGTG	sequencing
	M13R	GGAAACAGCTATGACCATG	

### Cleaning and clustering

The sequences were cleaned using Seqclean (http://www.tigr.org) with a local vector database and default parameter settings, to remove vector, poly (A) and short sequences below 100 nt. EST sequences representing contamination from bacterial, yeast or fungal sources were identified using blast search and removed before further analyses. The dataset was clustered using cluster (http://genome.uiowa.edu/pubsoft/software.html), and assembled sequences were constructed by Phrap (http://www.phrap.org/phredphrapconsed.html) using default settings, generating contigs (clustered ESTs) and singletons (non-clustered ESTs), commonly referred to as “unigenes”.

### Sequence analysis

A BLASTX search was performed with all unigenes of *D. destructor* against the NCBI Nr dataset. BLASTN searches were performed against the NCBI nucleotide database and BLASTP and TBLASTN searches were done against the genomes of *M. incognita, M. hapla*, *Bursaphelenchus xylophilus*, *Globodera pallida* and *Heterodera glycines*. A TBLASTX search was used against ESTs of 20 species of plant-parasitic nematodes. The BLAST results were classed using a perl script. In addition, *D. destructor* unigenes were used to search against the model species *Caenorhabditis elegans* (Wormpep v.234) and *Brugia malayi*. The cut-off for sequence similarity was E-value<10^−5^ for all blast searches.

### Translation into putative proteins

The open reading frames (ORFs) of all identified unigenes were translated by the Genescan prediction and translation programs [31]. Signal peptide prediction was performed with SignalP 4.0 [32] and transmembrane domain prediction was performed by TMHMM (http://www.cbs.dtu.dk/services/TMHMM/).

### Functional assignments

BLAST2GO was used to map and annotate Gene Ontology (GO) terms [33], with default parameters, except for an E-value cut-off of 1e-6, maximum BLAST hits of 30, the conversion of the annotation to GOSlim view, and a node scoring filter in the GO graph of 50 for biological process, 20 for molecular function and 20 for cellular component. The returned results were classed and analyzed using a perl script and WEGO [34]. Further, as an alternative means of assigning function to clusters, clusters were also assigned to metabolic pathway using KOBAS to annotate KEGG [35]; assignments were made by requiring that the highest-scoring BLAST matches in SWIR V.21 have an assigned enzyme commission (EC) number.

### *C.elegans* homologues with RNAi phenotype

To identify cases where *D. destructor* and *C. elegans* share orthologous genes, which have been surveyed in *C. elegans* for knockout phenotype using RNAi, a local BLASTx search was used against the *C. elegans* protein database (Wormpep v.234). Results were searched for RNAi phenotype via WormMart section of WormBase (WS220).The sequences of these *C. elegans* genes with lethal RNAi phenotype were BLASTed against proteins database including plant (rice, maize and *Arabidopsis thaliana*) and human.

### *In situ* hybridization

Some candidate effectors were used for *in situ* hybridization to confirm the expression site as previously described [36], [37]. Subsequent linear PCRs were used to synthesize the probes with digoxigenin (DIG)-labelled dNTP (Roche, Mannheim, Germany). The sequences of primers are given in Table 1.

### Sequence bioinformatics analysis and phylogenetic tree reconstruction

The expansin proteins of *D. destructor* and similar sequences were aligned using the ClustalW algorithm in MEGA 5.0 [38]. Phylogenetic analysis of the sequences was performed by the workflow of the Phylogeny.fr platform [39], comprising sequence alignment by the MUSCLE software (v3.7) and removal of ambiguous regions by Gblocks (v0.91b) using the following parameters. The phylogenetic tree was reconstructed from the protein sequences using the maximum likelihood method implemented in the PhyML program (v3.0 aLRT). JTT was used as substitution model with four substitution rate categories; number of invariant sites and gamma distribution parameter were estimated. The gamma shape parameter was estimated directly from the data (gamma = 1.119). Reliability of internal branches was assessed using the aLRT test (SH-Like). Graphical representation and edition of the phylogenetic tree were performed with TreeDyn (V198.3) and MEGA 5.0 [38].

## Results and Discussion

### cDNA library construction and EST generation

ESTs were generated from a library constructed from mixed stages of *D. destructor* (Table 2). The cDNA insert lengths ranged in size from about 0.8 Kb to 3.5 Kb (not shown). 9800 high quality ESTs were obtained and used for further analysis. The average length of the ESTs was 477 nt and their average GC content was 47.9%. All sequences have been submitted to the dbEST of Genbank under accession numbers (JZ125157–JZ134956).

**Table 2:** cDNA library and ESTs summary of *D. destructor.*

Titre of cDNA library (pfu/ml)	1.4×10^6^
cDNA insert size(kb)	0.8–3.5
Total cDNA clones picked and sequenced	13237
Sequences passing quality check	9800(74%)
Average length of ESTs (nt)	477
Singletons	3606
Contigs	1402
Total number of unigenes	5007
Average length of unigene (nt)	507
Number of unigenes with ORF	4661
Number of unigenes without ORF	346

### Cluster analysis and transcript abundance

**Figure 1:**
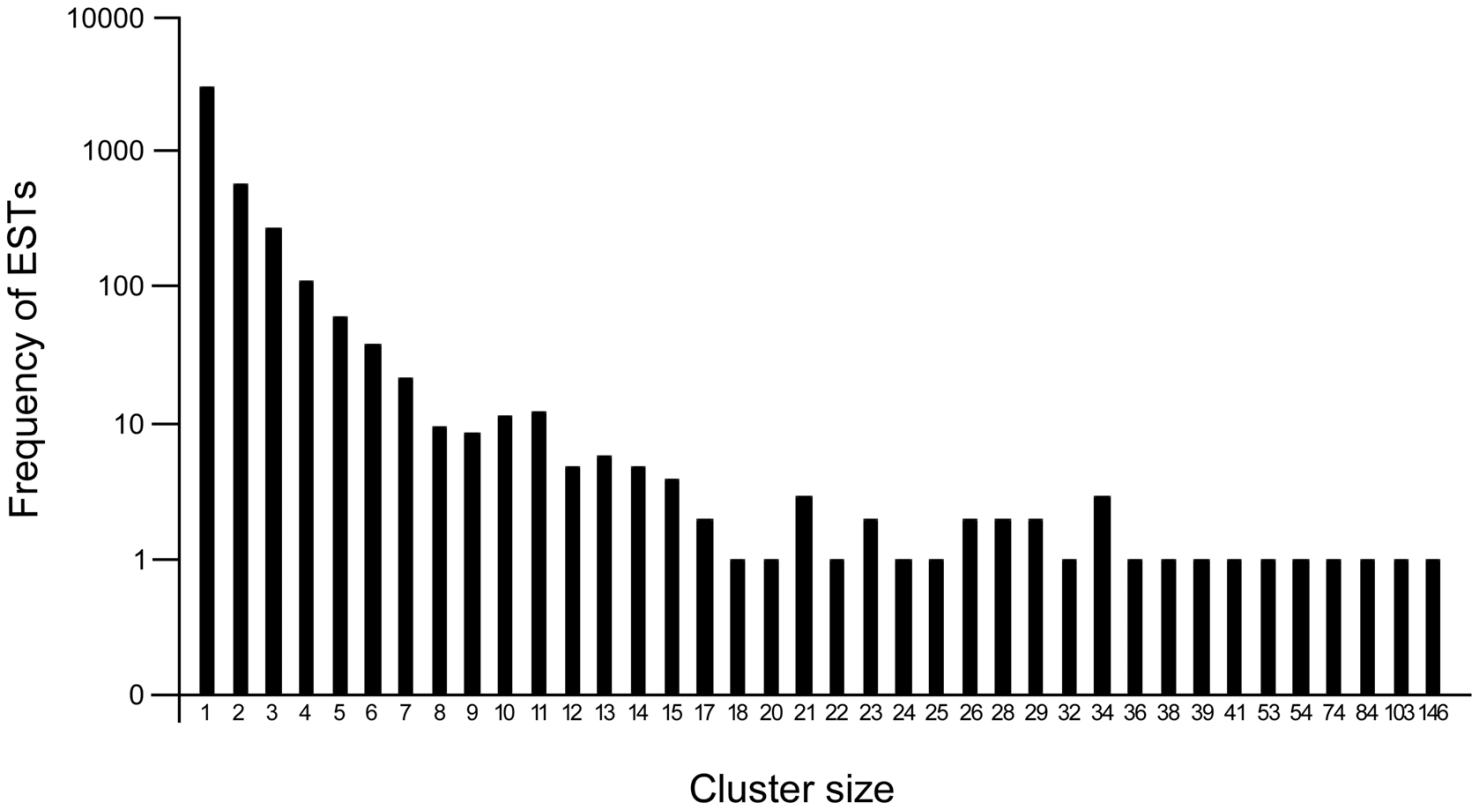
Distribution of clusters by number of assembles ESTs per cluster. Y axis, the number of clusters, X axis cluster size (i.e. the number of ESTs represented by the cluster).

The 9800 ESTs were classified into clusters by sequence identity. This analysis yielded 3606 singletons and 1402 contigs. These clusters varied from a single EST (for the 3606 singletons) to 147 ESTs (1 case) (Fig. 1). The top three single clusters account for 3.9% of the total ESTs, representing 146, 129 and 106 ESTs, respectively. Approximately 85.4% (1197 out of 1402) contigs were assembled by 2–5 ESTs. The average length of assembled transcript sequences was increased from 449 nt for submitted ESTs alone to 505 nt for the contigs. The longest sequence also increased from 746 to1531 nt. To the first approximation, the project has identified about 5008 clusters with a discovery rate for new genes of 51% (5008/9800). However, 5008 clusters were likely to be an over estimate of the true gene discovery rate, as one gene could be represented by multiple non-overlapping clusters. A certain degree of ‘fragmentation’ could be expected in our final dataset and was estimated by EST statistics to be as high as 15.8% using *C. elegans* as a reference genome [40]; another method described by Mitreva *et al.* [41] and resulted in a comparable estimation of 12.9%. After allowing for this fragmentation error (16%), we estimated that our dataset represents a minimum of 4206 genes. Assuming between 14,000 and 21,000 total genes, the range encompassed by *M. incognita* [23], *B. xylophilus* [24], *M. hapla* [25] and *C. elegans* (Wormpep v.234), the cluster dataset could represent approximately 20–30% of *D. destructor* genes.

The top 20 abundantly represented clusters contained 12% of ESTs (1163 of 9800), and each contained more than 26 ESTs (Table 3). 16 clusters had homologues with non-redundant database of NCBI and nematode ESTs. These transcripts primarily represented structural proteins and proteins involved in metabolic processes, such as actin, tropomyosin, aspartyl protease, cathepsin B-like proteinase, D-aspartate oxidase, FAD linked oxidase and UDP-glucuronosyl transferase. Other abundant sequences included three hypothetical proteins, two oxygenases and heat shock proteins. In addition, one of these transcripts represented by 79 ESTs encoded a putative endo-1, 4-gulcanase (ENG), suggesting that the ENG proteins are likely to play important roles in the parasitism and pathogenesis in *D. destructor*, as described for wide arrange of plant-parasitic nematodes [42]–[45]. Among the top 20 most abundant clusters, four clusters of transcripts had no similarity to any sequence in the databases of NCBI and five genomic sequence databases (*M. incognita*, *M. hapha*, *G. pallida*, *B. xylophilus* and *H. glycines*), suggesting that these genes may have specific functions in the development and parasitism of *D. destructor*.

**Table 3:** Top 20 abundant transcripts in the ESTs dataset of *D. destructor.*

No.	Cluster ID	ESTs	Top hit species and descriptor	Accession	E-value	%id
1	DDC05008	146	No hit (ORF lench282 bp)	—	—	—
2	DDC05005	129	beta-actin [*B. xylophilus*]	BAI52958.1	1.00E-76	62
3	DDC05003	106	ASpartyl Protease family member (asp-6) [*C. elegans*]	NP_505133.1	3.00E-37	64
4	DDC05002	86	beta-actin [*B. mucronatus*]	BAI52960.1	2.00E-68	77
5	DDC05000	79	GHF5 endoglucanase [*A. fragariae*]	AFD33558.1	2.00E-61	55
6	DDC04995	57	Hypothetical protein CBG21051 [*C.briggsae* AF16]	XP_001671832.1	3.00E-42	48
7	DDC04993	52	D-aspartate oxidase 1[*C. elegans*]	NP_504908.1	2.00E-50	50
8	DDC04998	51	Hypothetical protein CBG22199 [*C. briggsae* AF16]	XP_001667687.1	1.00E-12	33
9	DDC04991	50	No hit (ORF lench 192 bp)	—	—	—
10	DDC04989	43	tropomyosin fast isoform [*Ascaris suum*]	ADY44806.1	1.00E-42	62
11	DDC04988	42	UDP-glucuronosyltransferase ugt-48 [Ascaris suum]	ADY44031.1	7.00E-34	52
12	DDC04986	41	No hit (ORF lench279 bp)	—	—	—
13	DDC04985	38	4-hydroxyphenylpyruvate dioxygenase [Ascaris suum]	ADY45819.1	1.00E-54	80
114	DDC04984	36	FAD linked oxidase domain protein [*Dyadobacter fermentans* DSM 18053]	YP_003087689.1	7E-31	42
15	DDC04981	35	Hypothetical protein C04F12.8 [*C. elegans*]	NP_492580.1	3.00E-56	57
16	DDC04975	34	No hit (ORF lench285 bp)	—	—	—
17	DDC04972	31	heat shock protein HSP70 [P*leurodeles walt*]	Q91291.1	4.00E-88	88
18	DDC04997	30	Protein PGHM-1 [*C. elegans*]	NP_490898.1	6.00E-17	35
19	DDC04960	26	actin-2, partial [Wuchereria bancrofti]	EJW72694.1	2.00E-33	56
20	DDC04968	26	cathepsin B-like proteinase [Diabrotica virgifera virgifera]	XP_001897957.1	2.00E-16	46

### Comparative analysis of ESTs

**Figure 2:**
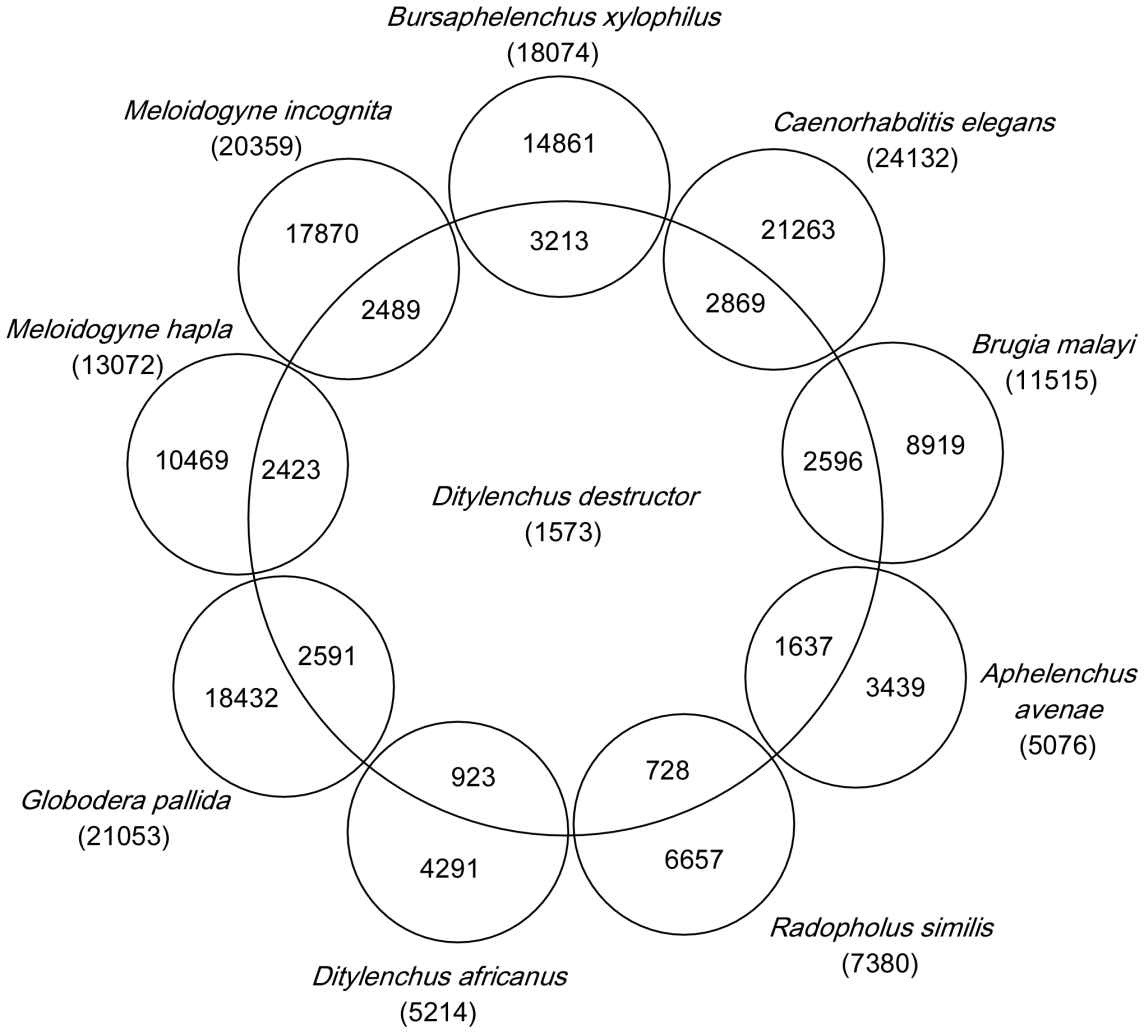
Comparison of *D. destructor* contigs with nematode genomes and ESTs. ESTs including *Ditylenchus africanus*, *Radopholus similis* and *Aphelenchus avenae*, Genomics data including *Brugia malayi*, *Caenorhabditis elegans*, *Bursaphelenchus xylopilus*, *Meloidogyne incognita*, *M. hapla* and *Globodera pallida*. Numbers in parentheses indicate total numbers of contigs.

Approximately 77% of unigenes of *D. destructor* (3618 of 5008) were similar to other known genes. In the majority of these cases (1244 of 3618), matches were present in all databases searched. Examination of the individual database searches showed that 3245 *D. destructor* sequences were similar to sequences in the NR database, 2869 in *C. elegans*, 2596 in *B. malayi*, 2489 in *M. incognita*, 2423 in *M. hapla*, 2591 in *G. pallida* and 3213 in *B. xylophilus* (Fig. 2). 1297 sequences have homologues in free-living (*C. elegans*), animal-parasitic (*B. malayi*) and plant-parasitic (*M. incognita*, *M. hapla*, *G. pallida* and *B. xylophilus*) nematodes, suggesting a role in general nematode development and metabolism. Meanwhile, 413 clusters had good matches exclusively in databases from other plant-parasitic nematodes, such as expansin [46], pectate lysases [47], endo-1,4-glucanase etc. The results implied that those genes may play important roles in parasitism and pathogenesis in plant-parasitic nematodes.

In addition, we found that 2862 clusters had similarities to the sequences in dbEST derived from other plant-parasitic nematodes, including 18.4% in *D. africanus*, 14.5% in *Radophilus similis*, 32.7% in *Aphelenchus avenae* and 37.2% in *B. xylophilus* (Fig. 2). The 15 gene products with the highest level of conservation between *D. destructor* and *D. africanus* are shown in Table 4. These include gene products involved in metabolism (cathepsin, phosphoenolpyruvate carboxykinase and phosphoribosyltransferase), protein biosynthesis and regulation (HSP90, elongation factor 1A, peroxiredoxin) and cell structure (ubiquitin, myosin and myosin regulatory light chain). In addition, 31 clusters of *D. destructor* were identified with no similarity to known genes except these of *D. africanus*. It is possible that these genes are specific to *Ditylenchus* spp. *D. africanus* is closely related to *D. destructor*, but the ESTs matched more homologues in *A. avenue* (n = 1638) and *B. xylophilus* (n = 1863) than in *D. africanus* (n = 923). We conjectured that the reason may due to the conditions in which the nematodes were cultured. *D. destructor*, *A. avenue* [12] and *B. xylophilus* [9] were cultured on fungi, whilst *D. africanus* was cultured on carrot [11]. Therefore, the results may not fully represent the phylogenetic relationship and the genome differences between the two nematodes. However, the results at least reflected the differences of gene expression between different culture methods.

**Table 4:** Top 15 conserved genes between *D. destructor* and *D. africanus*.

NO.	*D.destructor* clusters	*D.africanus* ESTs		NR database		
		EST No.	E-value	Accession	Top hit species and descriptor	E-value
1	DDC04778	DA03582	1.00E-122	ACT35690.1	cathepsin L-like cysteine proteinase [*D. destructor*]	0.0
2	DDC04012	DA02773	1.00E -120	AAL91109.1	ubiquitin [*Onchocerca volvulus*]	2.00E -134
3	DDC04897	DA00281	1.00E -118	DAA05871.1	eukaryotic translation elongation factor 1A [*H. glycines*]	3.00E -169
4	DDC04016	DA00057	1.00E -115	ADY43130.1	Phosphoenolpyruvate carboxykinase GTP [*Ascaris suum*]	6.00E -119
5	DDC04283	DA02680	1.00E -114	ADZ13510.1	HSP90-1 [*D. destructor*]	1.00E -126
6	DDC03687	DA05985	1.00E -113	EGT55546.1	CBN-EAT-6 protein [C. brenneri]	0.0
7	DDC04006	DA00450	2.00E -112	XP_001893253.1	hypothetical protein Bm1_08910 [*B. malayi*]	3.00E -26
8	DDC04942	DA04774	1.00E -105	XP_001892585.1	hypothetical protein Bm1_05555 [*B. malayi*]	1.00E -38
9	DDC03584	DA00032	1.00E -104	YP_676066.1	phosphoribosyltransferase [*Chelativorans* sp. BNC1]	3.00E -61
10	DDC00816	DA02886	2.00E -98	EJW77548.1	serine/threonine protein phosphatase 2A,partial [*Wuchereria bancrofti*]	1.00E -93
11	DDC03033	DA00221	2.00E -96	GAA55031.1	ubiquitin C [*Clonorchis sinensis*]	5.00E -104
12	DDC02696	DA01325	1.00E -94	ADY46489.1	SWI/SNF-related matrix-associated actin-dependent regulator of chromatin subfamily B member 1 [*Ascaris suum*]	1.00E -71
13	DDC04656	DA04271	3.00E -93	ADY47037.1	Phosphate carrier protein, partial [*Ascaris suum*]	9.00E -77
14	DDC02743	DA00825	7.00E -93	ADM35958.1	peroxiredoxin 1 precursor [*Haemonchus contortus*]	2.00E -67
15	DDC04847	DA04271	3.00E -91	AAL40718.1	myosin regulatory light chain [*M. javanica*]	8.00E -46

1391 clusters (27.8%) had no similarity to any sequences in the databases tested. These clusters had a shorter average sequence length (423 bp) than the unigenes that produced matches in the databases (536 bp). The sequences with unknown origin could correspond either to non-coding sequences (392 clusters without ORF), regulatory and structural RNA or to novel protein coding genes. Alternatively, they may be derived from 3′ untranslated regions and thus did not produce matches during similarity searches.

### Functional classification of ESTs

**Figure 3:**
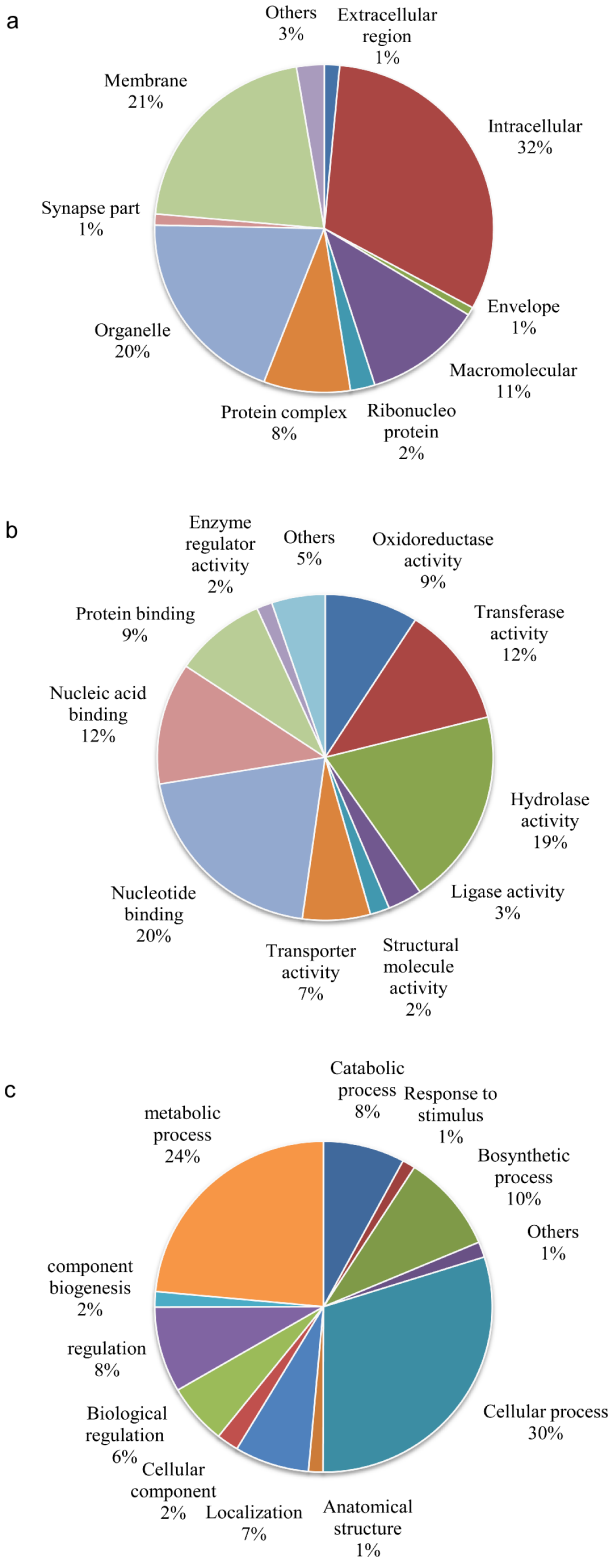
Assignment of Gene Ontology (GO) terms for clusters. Components including Cellular Component (A), Molecular Function (B) and Biological Process (C), are indicated. Individual GO categories have multiple mapping. Percentage reflects the total categories annotated but not the total sequences annotated under each component.

After mapping a total of 6561 gene ontology (GO) terms to the unigenes, 1991 clusters (39.8%) were annotated (Fig. 3). 1841 clusters generated matches in the Molecular Function class and retrieved 3768 GO terms, 1381 in the Biological Process with 1942 GO terms and 734 in the Cellular Component class with 851 GO terms. The highest GO term in the Molecular Function class was binding (GO: 0005488), including nucleotide binding (GO: 0000166) (20.2%), nucleic acid binding (GO: 0003676) (12.1%)), catalytic activity (GO: 003824) with hydrolase activity (GO: 0016787) (19.4%) and transferase activity (GO: 0016740) (12%). Within the Biological Process class, the cellular process (GO: 0009983) (29.9%), cellular metabolic process (GO: 0008152) (23.5%) categories were the most represented followed by biosynthetic process (GO: 0009058) (9.6%). For the Cellular Component class, the intracellular (GO: 0044424) (31.6%) and membrane (GO: 0016020) (20.9%) are the most highly represented (Table S1).

As an alternative method of categorizing cluster sequences by biochemical function, sequences were assigned to biological pathways using the Kyoto Encyclopedia of Genes and Genomes (KEGG) database (http://www.genome.ad.jp/kegg). 1550 clusters (31%) were assigned to enzyme commission (EC) numbers and 1211 clusters mapped to 181 KEGG biochemical pathways (Table 5). The KEGG pathways relating to metabolism were well represented by *D. destructor* sequences, including amino acid metabolism (n = 175), carbohydrate metabolism (n = 156), xenobiotics bio-degradation metabolism (88 enzymes), lipid metabolism (n = 86), energy metabolism (n = 76) and six other metabolism pathways (n = 207). About 13.7% of the unique sequences belonged to the genetic information processing (GIP) category. The KEGG pathways strongly represented in GIP are folding, sorting and degradation (n = 51), transcription (n = 37), translation (n = 30) and replication and repair (n = 44). Of these, 8% of the unique sequences belonged to the environmental information processing (EIP) category, indicating higher activities of stress and chaperone related genes during unfavorable conditions. The KEGG pathways strongly represented in EIP include signal transduction (100 enzymes), membrane transport (5 enzymes), and signaling molecules and interaction (4 enzymes). In addition 22.5% of sequences mapped to the cellular processes category. The immune system (n = 56), endocrine system (n = 49), cell growth and death (n = 49), transport and catabolism (n = 43), cell communication (n = 37) and other pathways (n = 66) were also the well represented categories in cellular processes (Table 4).

**Table 5:** KEGG biochemical mapping for *D. destructor* clusters.

KEGG pathway	clusters	Enzymes	Percentage (%)
**01100 Metabolism**	**862**	**641**	55.1
01101 Carbohydrate Metabolism	224	156	14.3
01102 Energy Metabolism	76	60	4.9
01103 Lipid Metabolism	92	70	5.9
01104 Nucleotide Metabolism	46	41	2.9
01105 Amino Acid Metabolism	175	134	11.2
01106 Metabolism of Other Amino Acids	46	38	2.9
01107 Glycan Biosynthesis and Metabolism	23	20	1.5
01108 Biosynthesis of Polyketides and Nonribosomal Peptides	2	1	<1
01109 Metabolism of Cofactors and Vitamins	45	33	2.9
01110 Biosynthesis of Secondary Metabolites	45	31	2.9
01111 Xenobiotics Biodegradation and Metabolism	88	57	5.6
**01120 Genetic Information Processing**	**214**	**162**	13.7
01121 Transcription	43	37	2.7
01122 Translation	53	30	3.4
01123 Folding, Sorting and Degradation	62	51	4.0
01124 Replication and Repair	56	44	3.6
**01130 Environmental Information Processing**	**137**	**109**	8.8
01131 Membrane Transport	22	5	1.4
01132 Signal Transduction	111	100	7.1
01133 Signaling Molecules and Interaction	4	4	<1
**01140 Cellular Processes**	**351**	**300**	22.4
01151 Transport and Catabolism	48	43	3.1
01141 Cell Motility	15	14	<1
01142 Cell Growth and Death	59	49	3.8
01143 Cell Communication	46	37	3.0
01150 Circulatory System	15	14	<1
01144 Endocrine System	63	49	4.0
01145 Immune System	62	56	4.0
01146 Nervous System	28	24	1.8
01147 Sensory System	4	3	<1
01148 Development	7	7	<1
01149 Behavior	4	4	<1

### RNAi phenotypes

*In planta* RNAi is one of the proposed strategies to control plant-parasitic nematodes [48], [49]. RNAi phenotypes were assigned to the unigenes on the basis of similarity with *C. elegans* proteins. 2869 unigenes from *D. destuctor* were similar to those of *C. elegans*. 2137 different *C. elegans* proteins were retained by removing redundant protein hits. With the 1442 unigenes (50.3%) having a *C. elegans* homologue, an RNAi phenotype was retrieved from Wormmart database. 71.8% the RNAi phenotypes (n = 1036) report a lethal effect. These sequences were compared with the proteins of plants (rice, maize and *Arabidopsis thaliana*) and human, resulting in 63 genes without similarity. While disruption of gene expression of these genes is expected to have a less profound influence on nematode survival, the strongest effects on nematode survival are expected when targeting genes involved in developmental processes. Therefore, these nematode-specific genes are potential candidates for parasitic control.

### Identification of candidate effectors

**Figure 4:**
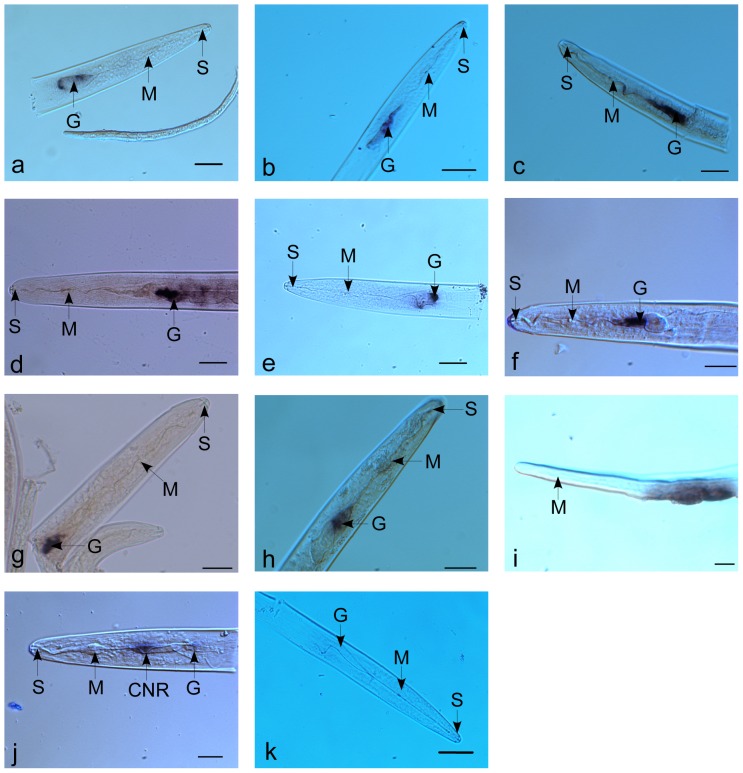
Localization of the effectors and expansin-like proteins from *D. destructor* using *in situ* hybridization. Section of the nematode incubated with antisense probe designed on putative effector genes: a, DDC05000(endo-1,4-beta-glucanase); b, DDC00149 (pactate lyase); c, *Dd-exp-1* from *D. destructor* ; d, *Dd-exp-2* from *D. destructor* ; e, DDC04837 (venom allergen antigen-like protein); f, DDC04927 (calreticulin protein); g, DDC02922 (annexin); h, DDC04384 (14-3-3b); i, DDC00459(flp protein); J, control. G, esophageal glands; S, stylet; M, metacorpus; CNR, circumpharyngeal nerve ring. Bar = 20 µl.

22 sequences with similarities to effectors from other plant-parasitic nematodes were identified within the ESTs, including a series of plant and fungi cell wall degrading enzymes (Table 6). Four ENG genes were represented in four different clusters. Three of them show greatest similarity to *A. fragariae* cellulase gene, while one has a higher similar to *D. africanus* cellulase gene. Two expansin-like proteins were identified with the best match to the expansin-like genes from *D. africanus*. One cluster has a high similarity with the pectate lyase gene of *A. avenae*, and was confirmed to be specifically expressed in the subventral pharyngeal gland cells of *D. destructor* by *In situ* hybridization (Fig. 4 a–d).

**Table 6:** *D. destructor* transcripts similar to effector genes.

Chusters	ESTs	Lenth (bp)	Nor redundant Genebank		
			Best hit description	Accession NO.	E-value
DDC04999	4	552	cellulase [*A. fragariae*]	AFI63769.1	4.00E-80
DDC04626	4	927	endo-1,4-beta-glucanase [*D. africanus*]	ACJ60676.1	3.00E-143
DDC05000	79	952	cellulase [*A. fragariae*]	AFD33558.1	3.00E-52
DDC04253	3	263	cellulase [*A. fragariae*]	AFI63769.1	1.00E-12
DDC03578	2	580	expansin-like protein [*D. africanus*]	ADJ57307.1	1.00e-63
DDC03579	2	541	expansin-like protein [*D. africanus*]	ADJ57307.1	2.00e-92
DDC00149	1	568	Pectate lyase [*A. avenae*]	BAI44499.1	3.00E-69
DDC04104	2	566	esophageal gland cell secretory protein 6[*H. glycines*]	AAG21336.1	9.00E-35
DDC04384	3	564	14-3-3b protein [*M. incognita*]	AAR85527.1	5.00E-52
DDC00830	1	183	calreticulin [*M. incognita*]	AAL40720.1	2.00E-19
DDC04255	3	591	calreticulin [*B. xylophilus*]	ADD82420.1	2.00E-71
DDC04927	14	633	calreticulin [*M. incognita*]	AAL40720.1	6.00E-68
DDC03093	1	495	VAP1 protein [*G. rostochiensis*]	CAD60978.1	2.00E-17
DDC04838	4	620	VAP1 protein [*G. rostochiensis*]	CAD60978.1	4.00E-19
DDC04839	4	696	VAP1 protein [*G. rostochiensis*]	CAD60978.1	9.00E-26
DDC03397	1	445	VAP-2 [*D. destructor*]	ADC35399.1	2.00E-5
DDC03835	2	607	SEC-2 protein [*Globodera pallida*]	CAA70477.1	5.00E-40
DDC02922	1	404	annexin [*B. xylophilus*]	ACZ13330.1	3.00E-64
DDC00061	1	516	chitinase domain-containing protein 1 [*Ascaris suum*]	ADY45342.1	4.00E-36
DDC01466	1	566	probable chitinase 2-like [*Bombus impatiens*]	XP_003492468.1	9.00E-41
DDC02490	1	315	endo-1,3(4)-beta-glucanase [*Exophiala dermatitidis*]	EHY54200.1	4.00E-22
DDC00721	1	441	endo-1,3-1,4-b-glucanase [*Metarhizium anisopliae*]	EFZ01017.1	1.00E-31

In the *D. destructor* ESTs, we found two clusters have significant similarity to 1, 3(4)-beta-glucanase protein of the GHF16 family. 1,3(4)-beta-glucanase genes had been also identified in the fungi-eating plant-parasitic nematodes *Bursaphelenchus* spp.[50], *A. avenae* [12] and *P. coffeae* [23]. There are two ESTs similar to the chitinase genes of fungi and *Ascaris suum*, but they do not match to the reported chitinase gene of plant-parasitic nematodes. Chitinases cleave the 1, 4-glycosidic bonds of chitin and have been found in a wide range of nematodes [9], [12], [51]. Since the chitin and glucan the major structural polysaccharides of the fungal cell wall, chitinases and beta-1, 3-glucanase are important in the breakdown of fungal cell walls and may facilitate feeding on fungi for nematodes like *D. destructor*, *Bursaphelenchus* spp. and *A. avenae*. However, both of the genes have not been found in another fungal feeding plantparasitic nematode, *D. africanus* [11]. One possible reason is that *D. africanus* was cultured on plants, and the expressions of genes relative to fungal substrates were not induced.

Several *D. destructor* ESTs are the homologues of other reported effectors. Included are venom allergen antigen-like protein, calreticulin, annexin, 14-3-3b, pharyngeal gland cell secretory protein and Sec-2. By in *situ* hybridization, the expressing patterns of venom allergen antigen-like protein was restricted to the subventral gland cells of *D. destructor* (Fig. 4 e) and calreticulin, annexin and 14-3-3b were expressed in the dorsal gland cell (Fig. 4 f–h). Venom allergen antigen-like protein, calreticulin and annexin have been reported as the key effectors in plant defense suppression and play an important role in nematode infection success [52]–[54]. 14-3-3b protein expressed in the dorsal gland of infective second-stage juveniles (J2) has been identified as being present in the secretome of *M. Incognita*. This gene may involve in guide protein-protein interactions and has essential roles in hormonal signal transduction processes in plants[55].

The full EST dataset was analyzed for putative secreted proteins with signal peptides but without a transmembrane domains [56]. The results showed that 634 clusters had signal peptides and 391 of these had no transmembrane domain. 191 of these proteins (47%) were found to have significant similarity to known sequences at NR database. Most of candidate effectors of *D. destructor* were present in this list except 14-3-3b, and over 50 clusters were termed as hypothetical proteins. 200 proteins have no significant similarity with known proteins; these may include novel effectors and their functions needs to be confirmed in the future (Table S2). We also found the transcript of a putative secreted protein that encodes a putative FMRFamide-like peptides (DDC00459) that is enriched in the circumpharyngeal nerve ring of *D. destructor* (Fig. 4 i).

### Characterization of two expansin genes

Expansin proteins secreted by plant-parasitic nematodes loosen plant cell wall components, and have been functionally characterized in several plant-parasitic nematodes [57]–[60]. In *D. destructor* ESTs, two expansin genes were identified, and named as *Dd-exp-1* and *Dd-exp-2*. *Dd-exp-1* has highest similarity to the expansin gene of *D. africanus*, while *Dd-exp-2* has highest similarity to the expansin gene of *B. xylophilus*. The full length cDNAs of *Dd-exp-1* and *Dd-exp-2* were obtained by rapid-amplification of cDNA ends (RACE).

**Figure 5:**
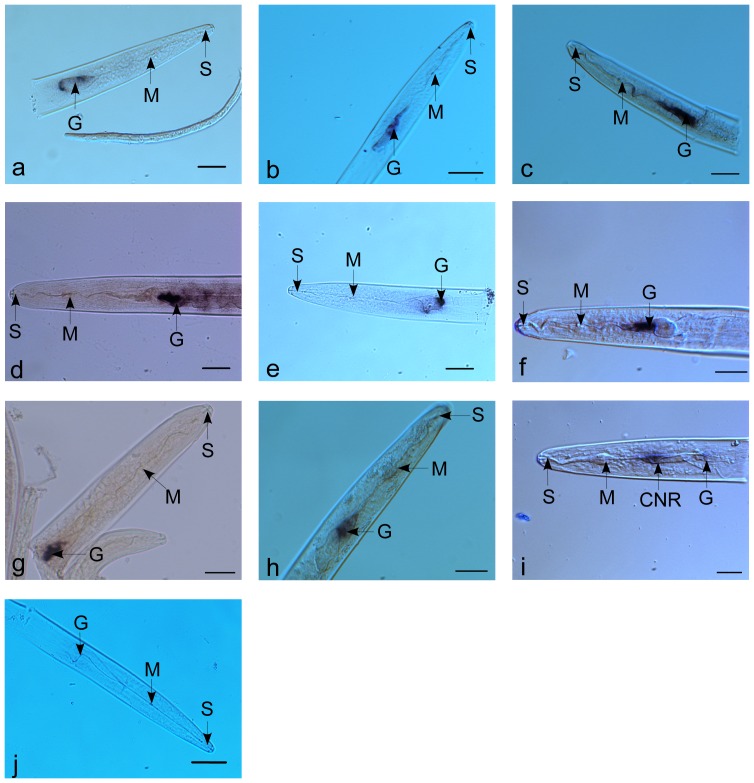
Multiple sequence alignment of DD-EXP-1 and DD-EXP-2 expansin proteins from *D. destructor* with that of other plant parasitic nematodes and plant. Residues identical to those of DD-EXP-1 and DD-EXP-2 are shaded black and similar residues are shaded grey. The conserved cysteines (C) in the expansin (like) family indicated with C1 to C6, the conserved cysteines in the expansin (like) proteins from plant parasitic nematodes are indicated by an asterisk(*). The positions of the introns are marked by D1 in DD-EXP-1 and DD-EXP-2, D2 in DA-EXP-1, H in HA-EXPB-1, B in BX-EXP-1 and BM-EXP-1, and G in GR-EXP-1. White triangles, black triangles and black diamond represent phase 0, 1 and 2 introns, respectively. Da, *Ditylenchus africanus*; Bx, *Bursaphelenchus xylophilus*; MJ, *Meloidogyne javanica*; MI, *M. incognita*; MH, *M. hapla*; HG, *Heterodera glycine*; HA, *H. avenae*; GP, *Globodera pallida*; GR, *G. rostochiensis*. PPAL [AAG52887] from *Nicotiana tabacum*.

The full length cDNA of *Dd-exp-1* (Genbank accession No. GU373911) is 1,207 base pair (bp) with a 930 bp open reading frame (ORF). The ORF encodes 309 amino acids with a putative ATG start codon at position 166 and TAG termination codon at position 1,096. The full length cDNA of *Dd-exp-2* (accession number GU373912) is 1,114 bp in length and contained a 900 bp ORF encoding 300 amino acids with an ATG start codon at the position 33 and a TAG stop codon at the position 936. The genomic DNA of *Dd-exp-1* and *Dd-exp-2* were 1,433 bp and 1,437 bp long from the start codon to the stop codon, respectively. The intron analysis showed that both contained one short intron (64 bp and 88 bp, respectively) and one longer intron (440 bp and 446 bp, respectively). The splicing sites of the introns followed the canonical ‘GU-AG rule’. The positions of the two introns of *Dd-exp-1* were identical to those of *Dd-exp-2*. Moreover, the intron positions and numbers within both *Dd-exp-1* and *Dd-exp-2* were the same to that in *Da-exp-1*(GU129695) from *D. africanus*. However, each of *Bx-exp-1*, *Bx-exp-2*, *Bm-exp-1* and *Bm-exp-2* from *Bursaphelenchus* spp. only has one intron located at the same position as the *Dd-exp-1* and *Dd-exp-2*. Both *Gr-expb1* (AJ556781) from *G. rostochiensis* and *Ha-expb-1* (JN861113) from *H. avenae* have five introns, and the intron positions and phases are completely consistent between these sequences. *Gr-expb1* and *Ha-expb-1* share one intron position (the fifth) with *Dd-exp-1* and *Dd-exp-2* (Fig. 5).

The molecular weights of putative DD-EXP-1 and DD-EXP-2 proteins were 30.44 kDa and 31.47 kDa and the theoretical pI value were 4.88 and 5.05, respectively. Both DD-EXP-1 and DD-EXP-2 proteins had signal peptides for secretion with the most likely cleavage site between amino acids 19 and 20. The conserved domain search confirmed that the DD-EXP-1 and DD-EXP-2 contained two distinct domains, an expansin domain (amino acid region 20-147 for both proteins) coupled to a bacterial type cellulose-binding domain (amino acid region 222–309 for DD-EXP-1 and 212–300 for DD-EXP-2). DD-EXP-1 and DD-EXP-2 has 75% and 73% identity with the DA-EXP-1 from *D. africanus*, respectively. In addition, DD-EXP-1 and DD-EXP-2 also had certain degrees of similarities to various hypothetical or unknown proteins from different microorganisms, and the pathogenicity or virulence factor precursors from *Meloidogyne* spp. with approximately 33% identity. Moreover, the CBD domain of DD-EXP-1 shared 62% and 63% identity with the CBMs in endo-1,4-glucanases from *D. destructor* and *D. africanus* (*Dd-eng-1a*, *Dd-eng-1b* and *Da-eng-1*), respectively.

Searching EST datasets from twenty nematode species with the expansin domain of DD-EXP-1 resulted in the identification of one matching contig from *D. africanus*, *H. glycines*, *B. mucronatus*, *B. xylophilus* and *P. vulnus* separately, two matching contigs from *Xiphinema index*, three matching contigs separately from *M. paranaensis* and *G. rostochiensis*, four matching contigs separately from *M. arenaria* and *M. chitwoodi*, five matching contigs separately from *M. incognita* and *M. javanica*, and eleven matching contigs from *M. hapla*. In addition, we conducted BLASTP searches on the predicted protein databases from *M. incognita*, *M. hapla*, *B. xylophilus* and *G. pallida* using the expansin domain of DD-EXP-1. Several expansin homologous proteins were identified from the four species of plant-parasitic nematodes (Table S3).

**Figure 6:**
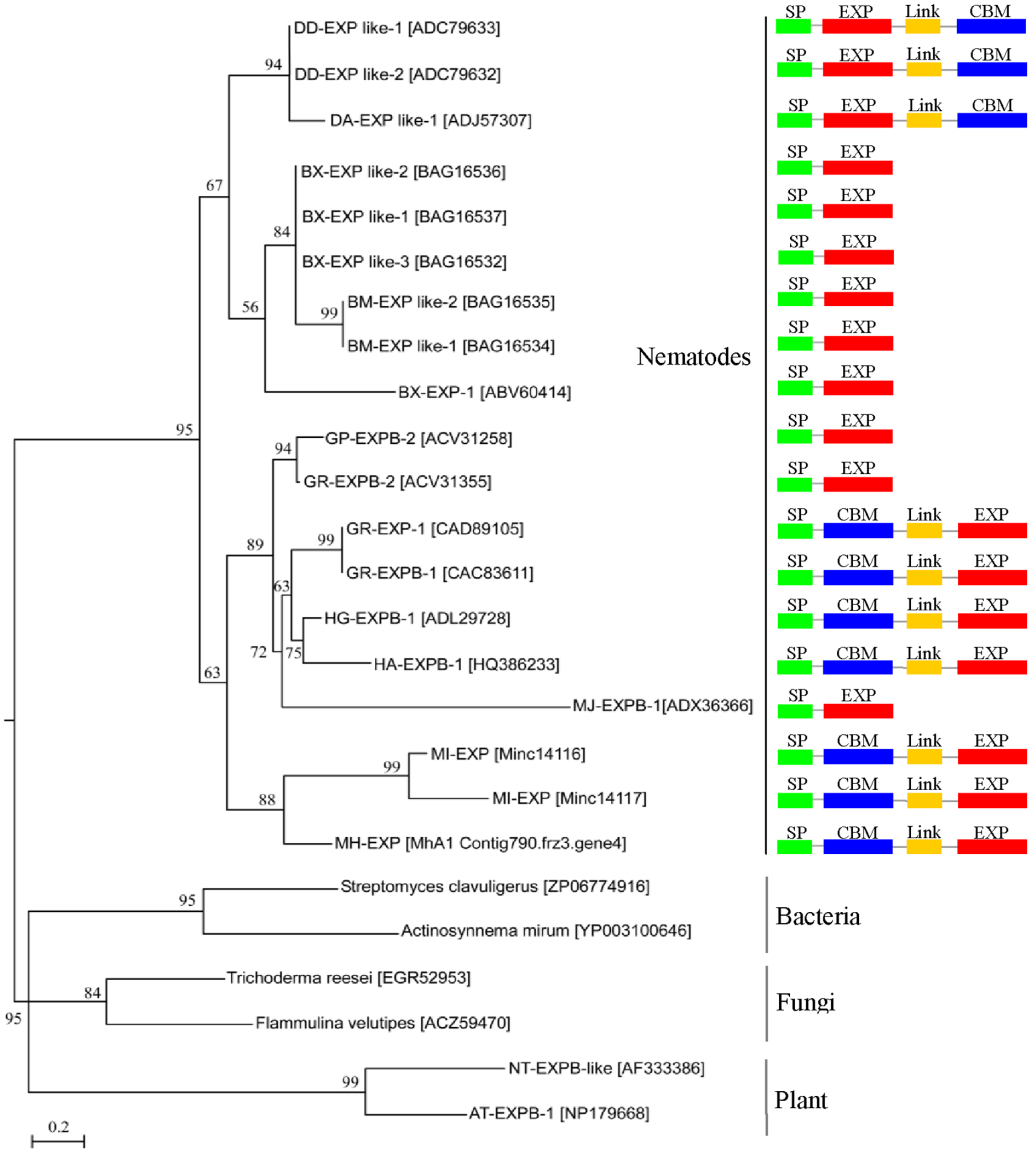
Phylogenetic tree of selected expansin proteins generated using maximum likelihood of PhyML. Numbers next to the branches indicate the bootstrap value calculated from 1000 replicates. The Genbank accession number of expansin proteins from *D. africanus* (Da-EXP-1), *B. xylophilus* (BX-EXPB-1, BX-EXPB-2, BX-EXPB-3, BX-EXP-1), *B. mucronatus* (BM-EXPB-1, BM-EXPB-2), *G. rostochiensis* (GR-EXPB-1, GR-EXPB-2 and GR-EXP-1), *G. pallida* (GP-EXP-2B) , *H. avenae*(HA-EXPB-1), *H. glycines* (HG-EXPB-1), *M. javanica* (MJ-EXPB-1) and the prediction proteins of genomes from *M. incognita* (MI-EXP), *M. hapla* (MH-EXP), bacteria, fungi and plant are indicated in brackets.

Expansin proteins from plant-parasitic nematodes were classified into three groups according to the domain structure (Fig. 6). In Group I, the expansin domain had a linker sequence and a cellulose binding domain (CBD) at the C terminal, and the sequence of the domains as signal peptide-expansin domain-linker-CBD. DD-EXP-1, DD-EXP-2 and DA-EXP-1belonged to Group I. In Group II, the expansin proteins contain a signal peptide and an expansin domain without CBD. BX-EXPB-1, BX-EXPB-2, BX-EXPB-3, BM-EXB-1, BM-EXB-2 belong in Group II [23]. In Group III expansin-like proteins had a signal peptide followed by a cellulose binding domain at the N terminal that connected to an expansin-like domain by a linker sequence. HG-EXP-1, HA-EXP-1, GR-EXP-1, GR-EXPB-1, MH-EXP and MI-EXP were included in Group III. In the genome sequences of *M. incognita*, *M. hapla*, *B. xylophilus*, and *G. pallida*, there are two copies of expansin gene sequences containing a N-terminal CBD and an expansin-like domain. According the current evidences, expansin proteins from the cyst and root-knot nematodes were classed into Group II and Group III. The expansins of the pine wood nematode, *B. xylophilus*, and *P. coffeae* were in Group III, and the expansins of *D. destructor* and *D. africanus* were found in Group I.

Multiple sequences alignment of plant-parasitic nematodes and plant species indicated that the presence of a series of conserved cysteines marked as key signatures of the expansin family [60]. Three of the six cysteines were conserved in both plants and plant-parasitic nematode expansin proteins. Two other cysteines were well conserved in all plant-parasitic nematode expansin proteins (Fig. 5). Another key motif of the plant expansin family is the HFD motif [60], but only the Hand F residues are conserved in the expansin proteins of plant-parasitic nematodes. In most of these sequences, the F is changed into one of the aliphatic amino acids (V, I, L or A). Similar conservation as well as an F to V conversion was found in the GR-EXPB1 from *G. rostochiensis*, for which the cell wall expansin activity on the type II primary cell walls was confirmed [46], [58]. This indicated that the amino acid change from F to V or other conversion does not affect the typical activity of expansin protein.

A phylogenetic tree was generated for the expansins from nematodes, plants, bacteria and fungi (Fig. 6). DD-EXP-1 and DD-EXP-2 were clustered together with the expansins from *D. africanus* and *Bursaphelenchus* spp. The root-knot nematode and cyst nematode expansins were clustered in other clades. The results indicated that the expansins from *Ditylenchus* spp. are closer to the expansins from *Bursaphelechus* spp. than to the expansins from the cyst and root-knot nematodes. Moreover, the identical position and phase of the shared introns in expansin domain suggested that the expansins from a wide range of plant-parasitic nematodes may have a common origin. The ancestral expansins of almost all plant-parasitic nematodes may only contain the expansin domain and was probably derived from horizontal gene transfer [61].

The tissue localization of DD-ENG-1 and DD-EXP-2 were assayed in *D. destructor* by in *situ* mRNA hybridization (Fig. 5c–d). The digoxigenin-labelled antisense cDNA probe hybridized specifically with transcripts accumulated within the two subventral pharyngeal gland cells of *D. destructor*. No hybridization signal was observed in sections with a control probe. Similar expression profiles have been described for expansins from other plant-parasitic nematodes [46], [57], [59], indicating that the reported expansins from the plant parasitic nematodes may share a similar function during infection of a plant by the nematode.

Expansins are known to weaken non-covalent interactions between cellulose and hemicellulose polymers thereby inducing plant cell wall extension [62]. The expansin proteins in *D. destructor* have a predicted signal peptide at the N-terminal, plus their expression in the subventral pharyngeal gland cells suggested that the DD-EXP-1 and DD-EXP-2 proteins were most likely secreted into the host tissues by *D. destructor*. The phylogenetic analysis together with the identical position and phase of the share intron in expansin domain from plant-parasitic nematodes suggested that the expansins of plant-parasitic nematodes may have a common ancestor. In addition, the expansin proteins from plant-parasitic nematodes contain three different protein domain variants, implying that expansins are likely to have evolved several times during the nematode and plant interaction.

## Conclusions

We have described the first detailed molecular analysis of the important plant-parasitic nematode *D. destructor* by a systematic characterization of ESTs. 9800 ESTs were obtained from a mixed stage cDNA library of *D. destructor* and over 5008 unigenes were identified. Among unigenes, 1391 specific to *D. destructor* and 31 specific to *Ditylenchus* spp. had no similarity to any known genes. In addition, 22 sequences similar to published nematode effectors and 391 secretome members were identified from *D. destructor* ESTs. *In situ* hybridization assays revealed that the most abundant effectors were localized in the pharyngeal gland cells.

Although the number of ESTs analyzed in this study is not large enough to represent the whole profile of the *D. destructor* transcriptome, the set of identified and validated genes, which are involved in plant and fungi cell wall degradation or modification, supported the fact that *D. destructor* can either be a plant parasite or a fungal feeder. This is the first report of 1, 3-beta-glucanase in nematode species of Anguinae (Nematoda: Tylenchida). Examining the phylogenetic evolution of the *D. destructor* expansin structures suggested that the expansin domain from plant-parasitism nematodes may have a common ancestor, but the evolution of expansin proteins in plant-parasitic nematodes involved domain shuffling.

In conclusion, the ESTs of *D. destructor* are not only useful in understanding the molecular biology of the sedentory and the migratory endo-parasitic nematodes , but also offer a direct molecular basis to distinguish *D. destructor* and *D. africanus*, which were previously described as the same species. In addition, this dataset also provides a resource for the upcoming whole genomic sequencing that will reveal the full complement of potential effectors present in *D. destructor*.

## Supporting Information

Table S1 Gene Ontology mappings for *D. destructor* clusters. Note that individual GO categories can have multiple mappings. To obtain the complete GO mapping, a node sequence filter in the GO graph was used: 50 for biological process, 20 for molecular function, and 20 for the cellular component.
(XLS)

Table S2 The potentially secreted proteins of *D. destructor.* TBLASTX searches (E<1e-5) of clusters of *D. destructor* against *D. africanus* ESTs and Nr databases.
(XLSX)

Table S3 The homologous expansins in other nematodes. BLASTX searches (E<1e-5) of expansin domain of DD-EXP-1 against NR databases, tBLASTX searches (E<1e-5) of expansin domain of DD-EXP-1 against EST database from twenty species of plant-parasitic nematodes and BLASTP searches (E<1e-5) of expansin domain of DD-EXP-1 against genome datasets of plant-parasitic nematodes (*B. xylophilus*, *M. incognita*, *M. hapla*, *G. pallida*)
(DOCX)
