# Correction: Exploring the Host Parasitism of the Migratory Plant-Parasitic Nematode *Ditylenchus destuctor* by Expressed Sequence Tags Analysis

**DOI:** 10.1371/annotation/7ee47d0b-f642-451c-a052-fbc4f3df6a98

**Published:** 2014-01-03

**Authors:** Huan Peng, Bing-li Gao, Ling-an Kong, Qing Yu, Wen-kun Huang, Xu-feng He, Hai-bo Long, De-liang Peng

Figure 5 is incorrect. Please view the correct Figure 5 here: 

**Figure:**
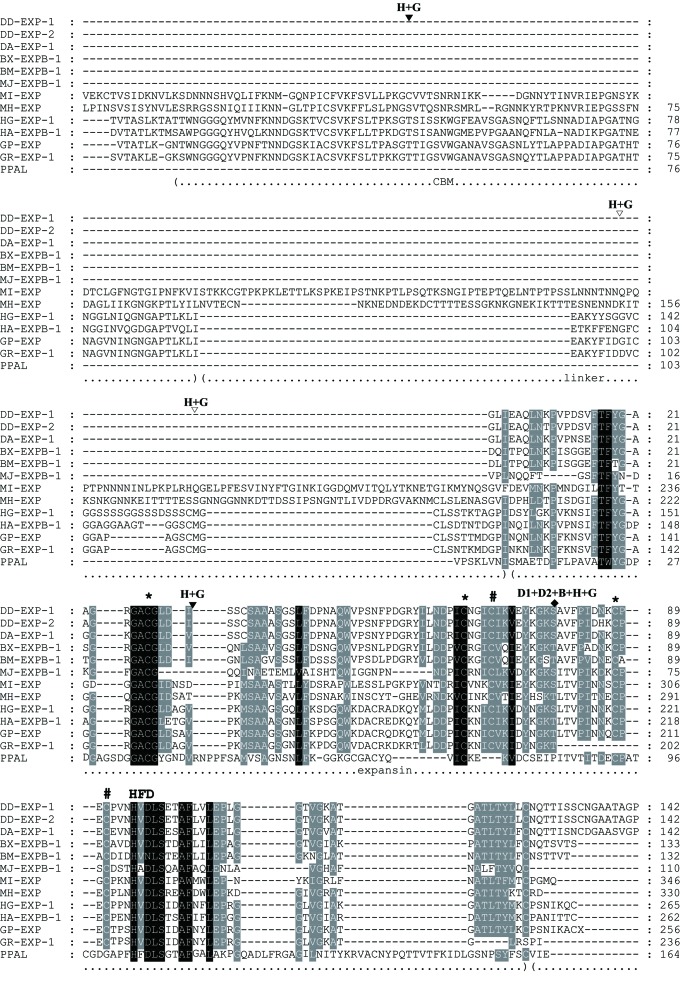


The title and legend of Figure 5 are correct. 

