# Supplementary material for: Exploring the Host Parasitism of the Migratory Plant-Parasitic Nematode Ditylenchus destuctor by Expressed Sequence Tags Analysis
Source: PLoS One. 2013 Jul 29;8(7):e69579. doi: 10.1371/journal.pone.0069579 (PMC3726699; doi:10.1371/journal.pone.0069579)
Supplement: Table S3 — The homologous expansins in other nematodes. BLASTX searches (E<1e-5) of expansin domain of DD-EXP-1 against NR databases, tBLASTX searches (E<1e-5) of expansin domain of DD-EXP-1 against EST database from twenty species of plant-parasitic nematodes and BLASTP searches (E<1e-5) of expansin domain of DD-EXP-1 against genome datasets of plant-parasitic nematodes (B. xylophilus, M. incognita, M. hapla, G. pallida) (DOCX) [file pone.0069579.s003.docx]

Additional file 3

The homologous expansins in other plant-parasitism nematodes

| **Species** | **Dataset** | **Accession No.** |
| --- | --- | --- |
| *Bursaphelenchus xylophilus* | Genbank | BAG16537.1, BAG16536.1, BAG16532.1, CAC84564.1 |
|  | ESTdataset | CJ986879, CJ981766, CJ985319 |
|  | Prediction proteins of genome | BUX_s01281.230.1, BUX_s01281.223.1, BUX_s01281.227.1, BUX_s01281.224.1, BUX_s01281.215.1, BUX_s01281.229.1, BUX_s01281.225.1, BUX_s01281.228.1 |
| *Bursaphelenchus mucronatus* | Genbank | BAG16535.1, BAG16534.1 |
|  | ESTdataset | CJ975871 |
| *Meloidogyne incognita* | Genbank | CAC27774.1 |
|  | ESTdataset | MI00559, MI01772, MI02719, MI05273, MI05733 |
|  | Prediction proteins of genome | Minc03214, Minc11468, Minc14599, Minc14116, Minc09177, Minc14117, Minc07960, Minc09488, Minc07955, Minc10989, Minc10988, Minc00158, Minc10366, Minc10365, Minc18856, Minc10987, Minc12725, Minc04469, Minc06816, Minc13768 |
| *Meloidogyne hapla* | ESTdataset | MH01015, MH02133, MH02764, MH02811, MH07368, MH07582, MH07601, MH07770, MH07998, MH08932, MH10906 |
|  | Prediction proteins of genome | MhA1_Contig2646.frz3.gene4, MhA1_Contig1015.frz3.gene7, MhA1_Contig790.frz3.gene4, MhA1_Contig1905.frz3.gene2, MhA1_Contig1905.frz3.gene1, MhA1_Contig1277.frz3.gene13 |
| *Globodera pallida* | Genbank | AEU04786.1, AEU04794.1, AEU04805.1, AEU04808.1, AEU04789.1, AEU04785.1, AEU04791.1, AEU04803.1, AEU04784.1, AEU04806.1, AEU04781.1, AEU04793.1, AEU04787.1, AEU04800.1, AEU04783.1, AEU04796.1, AEU04782.1, AEU04809.1, AEU04810.1, AEU04801.1, AEU04788.1, AEU04795.1, AEU04799.1, AEU04798.1, AEU04797.1, AEU04802.1, AEU04807.1, ACV31258.1, AEU04804.1 |
|  | Prediction proteins of genome | pathogens_Gpal_scaffold_169.g4107.t1, pathogens_Gpal_scaffold_59.g15454.t1, pathogens_Gpal_scaffold_169.g4108.t1, pathogens_Gpal_scaffold_8186.g19170.t1, pathogens_Gpal_scaffold_59.g15451.t1, pathogens_Gpal_scaffold_5333.g14320.t1, pathogens_Gpal_scaffold_166.g3987.t1, pathogens_Gpal_scaffold_143.g2834.t1 |
| *Globodera rostochiensis* | Genbank | AEU04821.1  AEU04823.1, AEU04820.1, AEU04826.1, AEU04819.1, AEU04829.1, AEU04831.1, AEU04833.1, AEU04830.1, AEU04839.1, AEU04841.1, AEU04832.1, AEU04824.1, AEU04840.1, AEU04842.1, AEU04835.1, AEU04834.1, ACV31355.1, ACV31351.1, ACV31366.1, AEU04818.1, AEU04837.1, ACV31300.1, AEU04838.1, ACV31277.1, AEU04817.1, ACV31362.1, ACV31304.1, ACV31333.1, ACV31270.1, ACV31350.1, ACV31345.1, ACN58322.1, ACV31262.1, ACV31293.1, ACV31297.1, ACV31325.1, ACV31308.1, ACV31367.1, ACV31251.1, ACV31257.1, ACV31271.1, ACV31311.1, ACV31260.1, ACV31368.1, ACV31331.1, AEE69368.1, ACV31319.1, ACV31340.1, ACV31324.1, ACV31248.1, ACV31289.1, ACV31252.1 |
|  | ESTdataset | GR11994, GR13760, GR13995 |
| *Globodera mexicana* | Genbank | AEU04814.1, AEU04812.1, AEU04815.1, AEU04813.1, AEU04816.1, AEU04811.1 |
| *Meloidogyne javanica* | Genbank | CAP59536.1, CAP59535.1, CAP59537.1, CAP59538.1, ADX36366.1 |
|  | ESTdataset | MJ00149, MJ00153, MJ00856, MJ01191, MJ04592 |
| *Ditylenchus africanus* | Genbank | ADJ57307.1 |
|  | ESTdataset | DA03457 |
| *Xiphinema index* |  | XI00223, XI00274 |
| *Meloidogyne paranaensis* | ESTdataset | MP00467, MP00635, MP00661 |
| *Meloidogyne arenaria* | ESTdataset | MA00256, MA00532, MA01696, MA03038 |
| *Meloidogyne chitwoodi* | ESTdataset | MC01139, MC01221, MC01774, MC01839 |
| *Pratylenchus vulnus* | ESTdataset | CV199817 |
| *Heterodera glycines* | Genbank | ADY02960.1, ADL29728.1 |
|  | ESTdataset | HG00639 |
| *Globodera tabacum* | Genbank | AEU04761.1, AEU04762.1, AEU04767.1, AEU04759.1, AEU04757.1, AEU04764.1, AEU04766.1, AEU04758.1, AEU04773.1, AEU04778.1, AEU04770.1, AEU04772.1, AEU04771.1, AEU04763.1, AEU04768.1, AEU04777.1, AEU04774.1, AEU04776.1 |
| *Aphelenchus avenae* | ESTdataset | GO480038 |
